# CRISPR-broad: combined design of multi-targeting gRNAs and broad, multiplex target finding

**DOI:** 10.1038/s41598-023-46212-x

**Published:** 2023-11-12

**Authors:** Alaguraj Veluchamy, Kaian Teles, Wolfgang Fischle

**Affiliations:** 1 Bioscience Program, Division of Biological and Environmental Sciences and Engineering, King Abdullah University of Science and Technology (KAUST), 23955-6900 Thuwal, Kingdom of Saudi Arabia; 2 Department of Computational Biology, St. Jude Children’s Research Hospital, Memphis, TN USA

**Keywords:** Computational biology and bioinformatics, Software, Epigenomics

## Abstract

In CRISPR-Cas and related nuclease-mediated genome editing, target recognition is based on guide RNAs (gRNAs) that are complementary to selected DNA regions. While single site targeting is fundamental for localized genome editing, targeting to expanded and multiple chromosome elements is desirable for various biological applications such as genome mapping and epigenome editing that make use of different fusion proteins with enzymatically dead Cas9. The current gRNA design tools are not suitable for this task, as these are optimized for defining single gRNAs for unique loci. Here, we introduce CRISPR-broad, a standalone, open-source application that defines gRNAs with multiple but specific targets in large continuous or spread regions of the genome, as defined by the user. This ability to identify multi-targeting gRNAs and corresponding multiple targetable regions in genomes is based on a novel aggregate gRNA scoring derived from on-target windows and off-target sites. Applying the new tool to the genomes of two model species, *C. elegans* and *H. sapiens,* we verified its efficiency in determining multi-targeting gRNAs and ranking potential target regions optimized for broad targeting. Further, we demonstrated the general usability of CRISPR-broad by cellular mapping of a large human genome element using dCas9 fused to green fluorescent protein.

## Introduction

The discovery of CRISPR-Cas and related RNA-guided nuclease systems has made a plethora of biological applications possible that require specific genome targeting^1,2^. In particular, the engineering of fusion proteins with enzymatically dead Cas9 (dCas9) has established new approaches for genome mapping as well as expanded the possibilities of genome manipulation beyond editing DNA sequence^3^. dCas9 fused to fluorescent proteins can, for example, be used to label specific chromosome elements such as telomeres and pericentromeres^4–6^. dCas9 targeting can further be used to directly control gene expression via promoter restricted recruitment of transcription factors such as shown with dCas9-VP64 activating endogenous genes in human cells^7^. Altering chromatin modifications (e.g., via fusing DNA methylation and histone modification enzymes to dCas9) holds high promise for modulating the epigenome and thereby establishing lasting but reversible genome manipulation^8–12^. Indeed, a dCas9 DNA methyltransferase 3A (DNMT3A) fusion protein was shown to install DNA methylation in the CpG island of the promoter region of a target gene thereby causing decreased expression^13^. Similarly, targeting histone methyltransferase systems dCas9-KRAB or dCas9-LSD1 to enhancer or other regulatory regions was found capable of modulating transcription^10,14^.

In the mentioned examples, the biological effects of dCas9 targeting were highly correlated to the number of single guide RNAs (sgRNA) recruited to the target region. It was shown that for dCas9 mediated mapping of telomeres and pericentromers more than 50 sgRNA mediated targeting events are needed^4,6^. Similarly, multiple sgRNAs were shown to be required for dCas9-DNMT3A to methylate a broad section of the genome for efficiently decreasing expression of IL6ST and BACH2 target genes^9^. Also, dCas9-KRAB mediated deposition of H3K9me3 on the HS2 enhancer required a panel of 21 independent sgRNAs to cover the entire 400 bp core of this regulatory element^15^.

Numerous tools have been developed for designing the gRNAs required for Cas9 genome targeting. Each of these has unique advantages and limitations^16^. While FlashFry, CRISPOR, CRISPR-DO and CasFinder are based on procedural approaches, some tools such as sgRNAScorer2, TUSCAN and CHOPCHOP adopt machine learning for target prediction^17^. One of the major concerns of the CRISPR-Cas9 and related approaches is off-target effects that are related to the number of mismatches in the gRNA protospacer sequences^18^. Several tools exist for the analysis of putative non-specific targeting of gRNAs. These generally fall into two classes, those that directly scan for sequence-based finding of off-targets (e.g. CasOT^19^, Cas-OFFinder^20^, and OffScan^21^) and those that make predictions of off-targets ( e.g. DeepCRISPR^22^ and CROP-IT^23^). The prevailing gRNA design tools are limited to defining gRNAs using a “one-gRNA-one target” approach and to reducing off-target effects. For targeting large regions of the genome, this requires defining multiple sgRNAs. CRISPR-Local screens for sgRNAs that can simultaneously target multiple genes but with singular binding sites^24^. CRISPR MultiTargeter can find common and unique CRISPR sgRNA in a set of similar sequences^25^. We have summarized the features of the different CRISPR gRNA design tools in Supplementary Fig. 1 and Supplementary Table 1. Essentially, none of the available tools is suitable for specifically targeting broad regions of the genome.

Targeting broader genome regions is generally possible by restricting the application to highly repetitive genome elements such as found in telomers or chromosome satellites (aka pericentromeres)^26^. Alternatively, multiple sgRNAs could be used. However, the latter approach increases the costs of experiments, raises the chances of off-target effects, requires multiple design rounds of gRNAs, and is subject to the limitations of introducing multiple targeting systems simultaneously in cells.

Motivated by these considerations, we set out to define an approach that solves this joined problem by introducing a multi-targeting gRNA-multiplex target design strategy. Our new, flexible tool CRISPR-broad can (i) identify and rank genomic regions that can be multi-targeted by single or few gRNAs (gRNA multiplex targets); (ii) specify single or few gRNAs that can target user-defined multiple and/or large genome regions (multi-targeting gRNAs), (iii) classify gRNAs that are highly dispersed for efficient targeting and manipulation of large genomic regions and (iv) define such gRNAs for different CRISPR-Cas related systems (e.g. CRISPR/Cas9, Cpf1/C2c1, etc.). In doing so, the program minimizes off-target effects.

## Results

### CRISPR-broad framework

**Figure 1:**
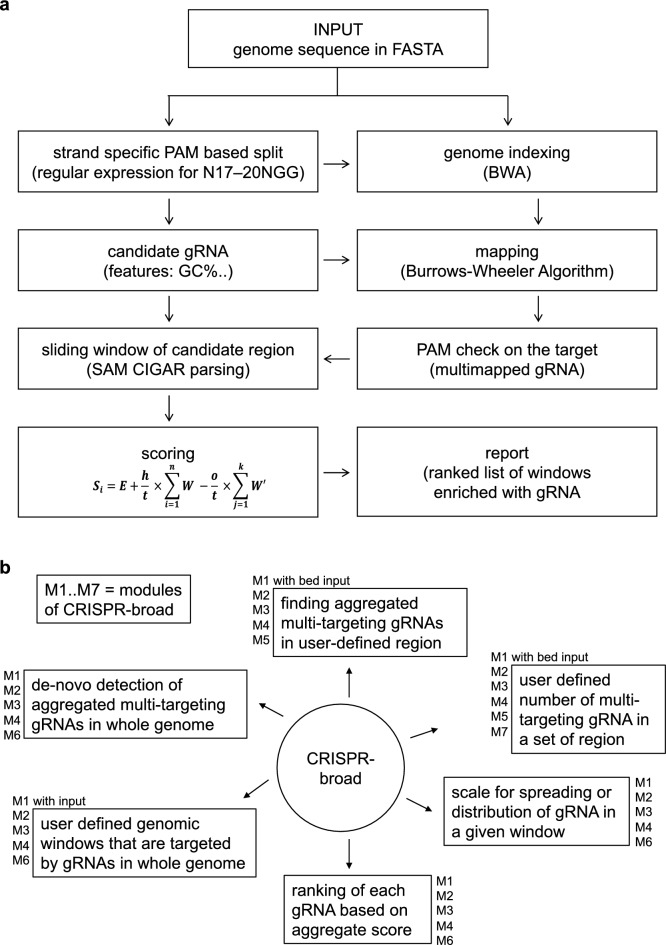
Modules and features of CRISPR-broad. (**a**) Working scheme of the CRISPR-broad tool. Several steps in this pipeline are multithreaded. The input is a multiFASTA genome file and each step can be individually executed. Indexing and mapping steps are time limiting and can be performed separately. The output of this pipeline is a ranked list of gRNAs in text format. (**b**) The different modules in execution of CRISPR-broad, their features and applicability as well as the respective options for user input are shown. The different options for running the individual modules are described in detail at https://github.com/AlagurajVeluchamy/CRISPR-broad.

We developed a procedural pipeline for detecting gRNAs and implemented this in Python as a standalone application (Fig. 1a). For speeding up gRNA selection, we employed multithreading and used big data Python module Pandas. This allowed splitting millions of short sequences for mapping and processing large numbers of uncompressed alignments. The different steps and options of CRISPR-broad are implemented in seven different modules (in Python with Pandas and PyRanges packages) to avoid re-performing steps that are computationally demanding. Multiple options for user input are available (Fig. 1b).

### *C. elegans* and *H. sapiens* genomes exhibit a wide range of gRNA selection possibilities for targeting broad regions

Running CRISPR-broad on the *C. elegans* genome (target window size 50 kb), we obtained 5,734,064 candidate gRNAs with the Cas9 PAM pattern NGG at the 3’-end and flanked by 20 nt at the 5’-end. We allowed a range of mismatches from 0 to 3 to map to the *C. elegans* genome assembly Ce235 using the “end-to-end” all alignment option in bowtie2. The large pairwise alignment was parsed for indels and matches to calculate a ranking score. About 18% of these candidate gRNAs were mapped to multiple sites. We further filtered entries that were aligned to less than five genomic loci. Our analysis resulted in 27,858 gRNAs (≥ five hits in the selected window) that could target 6421 unique 50 kb regions (Supplementary Fig. 2a).

**Figure 2:**
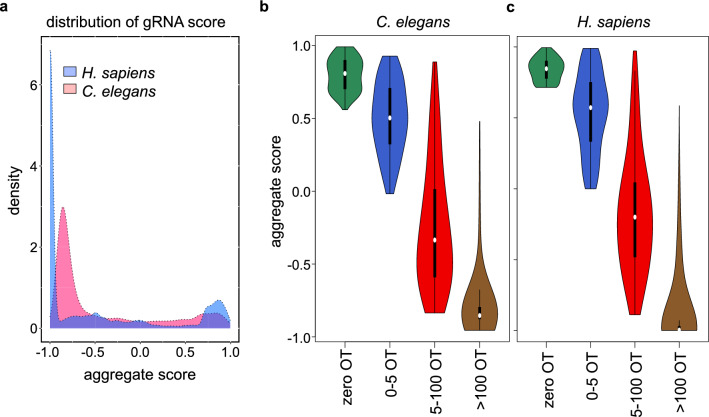
Aggregate gRNA score distribution for two model organisms. (**a**) The aggregate gRNA score ranging from − 1 to + 1 for two datasets is shown in a density plot. Aggregate gRNA score with up to 10 k off-target (OT) settings in *C. elegans* (**b**) and *H. sapiens* (**c**).

Next, we scanned the human genome (target window size 500 kb) and filtered candidate gRNAs with a cutoff of 50% GC. This resulted in around 120 million gRNAs. We mapped these sequences with a range of mismatches from zero to three and maximum hits of 10,000. The multi-mapped positions were verified for PAM sequences at their 3’-end and pooled. We processed candidate gRNAs further that had at least five hits in the genome. This combined filtering resulted in 2,413,602 (0.6%) gRNAs that target 1,678,629 windows (Supplementary Fig. 2b). The targetable windows with minimally five loci for a unique gRNA of the *C. elegans* and *H. sapiens* genomes were spread throughout the different chromosomes (Supplementary Fig. 2b). The aggregate gRNA score pattern distribution for both sample genomes showed that although off-targets are high (negative score), a significant number of high scoring regions in these genomes are available for gRNA targeting (Fig. 2a). Irrespective of genome size or sequence content, the aggregate score decreased with the number of off-targets thereby validating the score-based selection of gRNAs (Fig. 2b,c).

**Figure 3:**
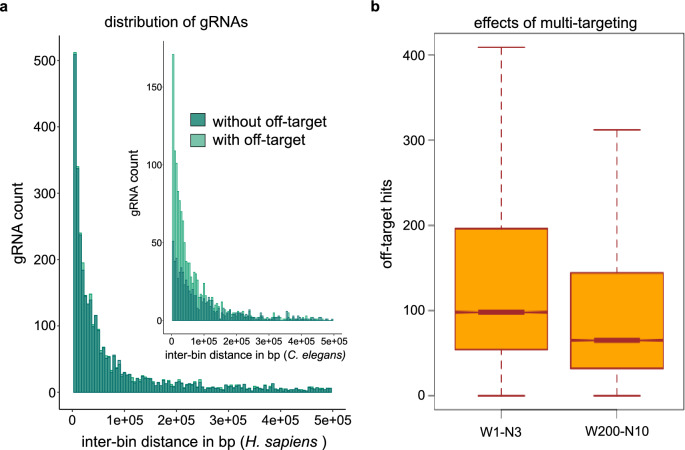
Distribution of gRNAs along the chromosomes of *C. elegans* and *H. sapiens.* (**a**) gRNA sequences clustered in small intervals are evident from this analysis on distribution of gRNA hits. Inter-bin distances of multi-hit gRNA sequences with and without off-target. The distances of gRNA hits are shown in bp (in equal bin size). Note the difference in the distribution of gRNAs with or without off-targets for *C. elegans* and *H. sapiens* due to the different repetitiveness of the two genomes. (**b**) Boxplot showing the relationship between size and number of target bins in the genome of *C. elegans*. Off-target hits represent the sum of gRNA hits that fall outside all the multiple target windows. W, window size; N, number of target windows.

Inter-bin distance defines the gap between two target regions and hence illustrates the density of target windows. Analysis of this parameter between different gRNA candidates with or without off-targets revealed that gRNA distribution is not biased over different chromosomes (Fig. 3a). Finding potential gRNAs was further supported by increase in window size and by selecting gRNA that are multi-targeting (Fig. 3b).

### Minimized potential off-targets

Typical unique sgRNA selection involves reducing off-target hits on multiple genomic regions and finding a unique target sequence. Tandem duplications in the genome are one cause of off-target effects. CRISPR-broad uses these duplication events in detecting gRNAs in bins (a large genomic region). Larger window sizes could reduce the potential off- target effect of gRNAs in our tool. This was evident from the number of on- and off-target hits (Fig. 3b).

**Figure 4:**
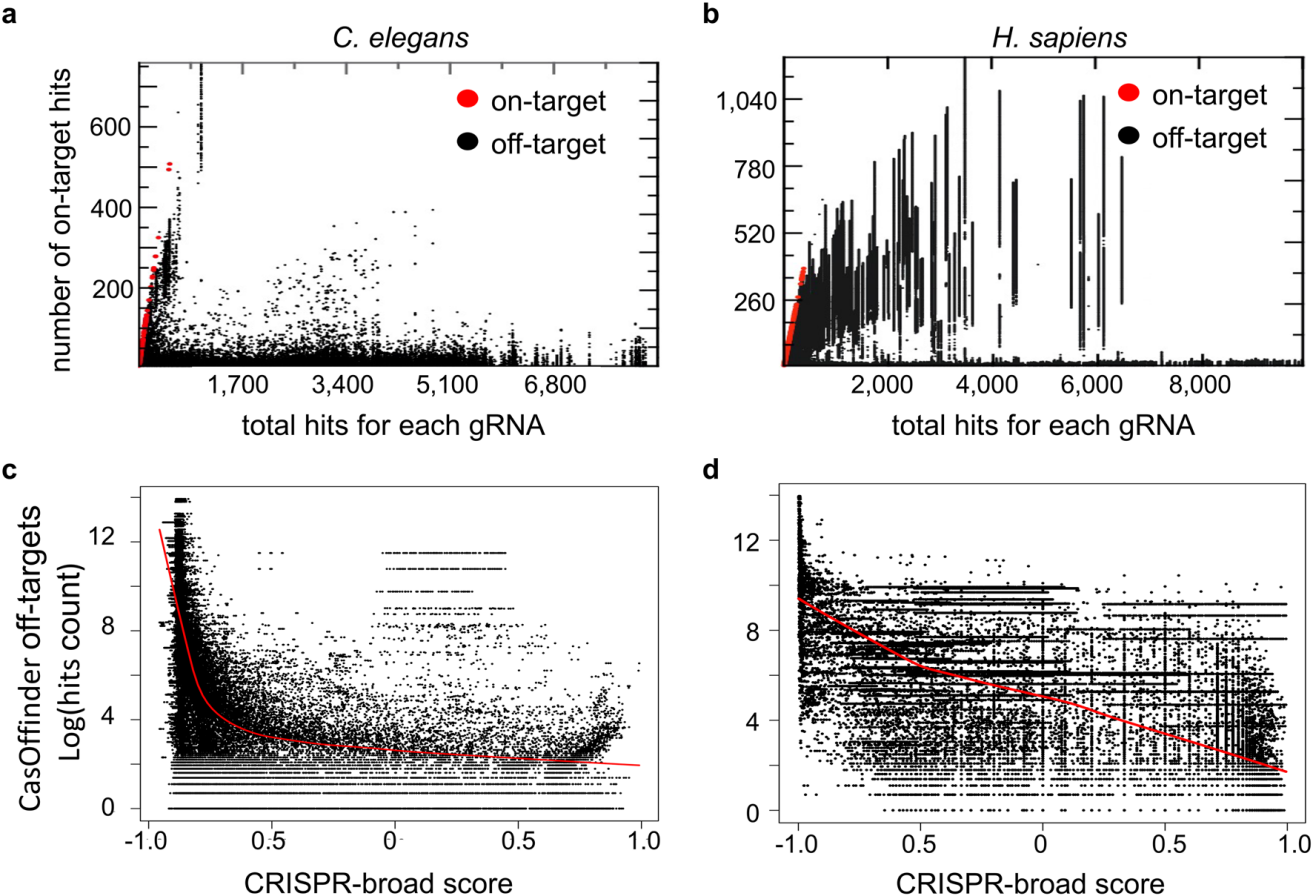
Relationship of on-target and off-target sites for each gRNA. Multi-hit alignment with short read aligner was performed for each gRNA. Number of hits within the selected window and off-target windows were enumerated from the alignment. (**a**) Off-target distribution in comparison to the number of on-target hits in *C. elegans* (50 kb window). (**b**) off-target distribution in comparison to the number of on-target hits in *H. sapiens* (500 kb window). (**c**) Off-targets predicted by CasOFFinder compared to the CRISPR-broad scoring system in *C. elegans*. (**d**) CRISPR-broad score for gRNAs in *H. sapiens* compared to off-targets predicted by CasOFFinder. The number of off-targets predicted for individual gRNAs is anticorrelated to our CRISPR-broad scoring system.

Each sgRNA has N total hits in the genome, T hits in the target window and O hits in the off-targets (region outside/different from the 50/500 kb target window). When analyzing the *C. elegans* and *H. sapiens* genomes, there was no correlation between N and O (Fig. 4a,b). The 50 kb and 500 kb windows showed a vast number of on-targets compared to off-targets, revealing a wide range of selectable regions. Indeed, on-target regions could be identified that showed a high number of gRNA loci with zero off-targets. This included a pericentromeric region of human chromosome 1, which has 272 gRNAs loci with no apparent off-targets (Supplementary Fig. 3a). Similarly, in *C. elegans* analysis with a window size of 10 kb revealed a region on the X chromosome (chrX:7351–7361 kb) where at least 1000 loci could be found for one gRNA (Supplementary Fig. 3b). The candidate target regions identified in both, *C. elegans* and *H. sapiens* were not limited to functionally annotated repetitive regions (e.g. telomeres, satellites) that could be directly targeted by classical gRNA design tools such as CHOPCHOP (Supplementary Fig. 3c,d).

Global comparison of the CRISPR-broad scores derived from analyzing the *C. elegans* and *H. sapiens* genomes to the results of an independent, state-of-the-art off-target scanning tool for individual gRNAs (CasOffinder), indicated that these are higher for gRNAs that were identified to have a lower number of predicted off-targets (Fig. 4c,d). This supported the notion that our scoring method is relevant for selection of multi-targeting gRNAs.

### Aggregate gRNA score based on on-target windows and off-target sites

We calculated cumulative scores for the gRNAs matching to selected loci and including a penalty score in case off-targets were found. These scores range from − 1 to + 1. In both genomes analyzed, *C. elegans* and *H. sapiens* we observed a bias towards the extreme values on both sides of the aggregate gRNA score, i.e. many gRNAs are either good candidates for multi-targeting with many hits and no off-targeting (aggregate gRNA score close to + 1) or are showing many off-target hits and mismatching (aggregate gRNA score of close to − 1) (Fig. 2a). The very high negative aggregate gRNA scores observed are reflection of repetitive elements such as Alu sequences, LINE-1 retrotransposons, MIR, and human endogenous retroviruses (HERVs), which represent 55% of the human genome, occurring in multiple copies^27^. Similarly, in the *C. elegans* genome MITE sequence repeats might elevate the number of off-targets^28^. These off-targets are correlated to the aggregate gRNA score (Fig. 2b,c).

**Figure 5:**
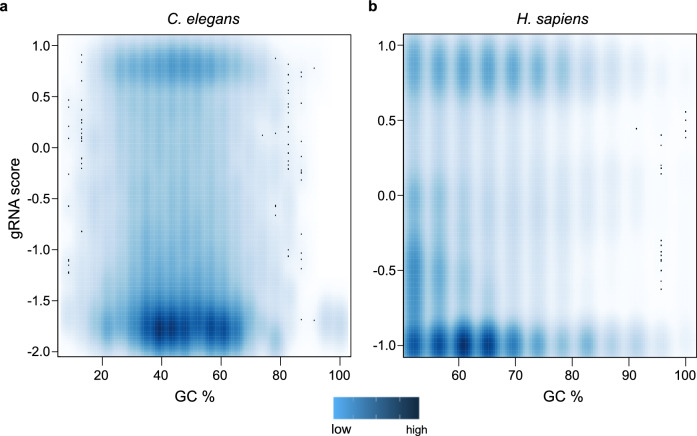
gRNA score correlation to GC composition of the 23 nucleotides gRNA sequence. (**a, b**) Sequence composition as dinucleotide frequencies were calculated. The gRNA score (range from − 2 to + 1) and the GC content are depicted in the density plot. Aggregate gRNA score and repetition of sequence (off-target) are independent of the sequence composition. Many candidate gRNAs with high aggregate gRNA score that corresponds to candidate target windows are available for varied GC content.

sgRNA efficiency has been correlated with the GC content of the nucleotide sequence^29^. We explored whether the GC content feature impacted the number of available gRNAs (with significant number of on-target hits and lower off-target hits). The aggregate gRNA scores (gRNA scores of each window) varied highly from the GC-contents of the sequences (Fig. 5). This indicated that CRISPR-broad scans a wide range of gRNAs that may have different levels of repetitive nucleotide sequences. The repetitive elements may be AT-rich and gRNA selection based on gRNA score is not limited by GC content.

### Increasing the size range and number of candidates increases potential aggregated multi-targeting

To elucidate the effects of user-defined bin size and number of distinct gRNA combinations, we scanned the *C. elegans* genome with two window sizes of 1 kb and 200 kb and targeting window numbers of 3 and 10. As expected, the number of off-targets decreased with increasing target window sizes and the number of target regions (Fig. 3b). Our analysis showed that with different bin sizes and using multiple gRNA, a wide range of regions can be selected for targeting with singular gRNAs.

### Assessment of displacement of gRNAs within on-target windows

**Figure 6:**
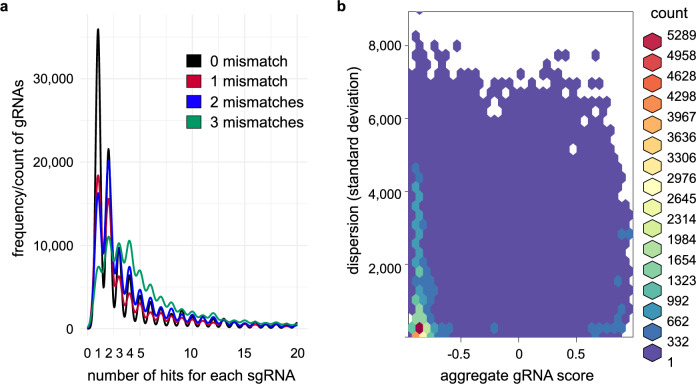
Assessment of displacement of gRNAs within on-target windows. (**a**) CRISPR-broad was used to scan for potential gRNAs with different levels of mismatches, since earlier reports have shown that the efficiency of gRNAs are limited by the number of mismatches. Mismatch levels and number of on-target hits for gRNAs of individual 50 kb windows in *C. elegans* are shown. Mismatch levels are set in the range from 0 to 3. Many selectable gRNAs and their corresponding target windows are available even at a mismatch level of 0. (**b**) Hexbin plot showing the relationship between aggregate gRNA score and dispersion. Standard deviation (dispersion) was calculated from the position of the gRNA hits within a target window. The aggregate gRNA score ranges from negative to positive values. Higher values of standard deviation correspond to higher distribution of gRNA within a target window. Standard deviation and gRNA score were calculated using 500 kb windows in *H. sapiens.*

The dispersion of a gRNA within a bin is depending on the number of hits and this increases with the number of mismatches (0–3). Nevertheless, most hits for gRNAs were unique with no mismatches. This is revealed from sgRNA mismatch analysis of the whole genome of *C elegans* and a random selection of 10,000 sgRNA in *H. sapiens* (Fig. 6a and Supplementary Fig. 5a). Also, these mismatches were independent of the position within a bin (Supplementary Fig. 4). Further, the dispersion of individual gRNAs did not correlate with the aggregate gRNA score in both *C. elegans* and *H. sapiens*. In *C. elegans* most gRNAs with higher standard deviation from the mid position of the bin showed lower aggregate gRNA scores (Fig. 6b). Also, in *H. sapiens*, the standard deviation was not correlated to the gRNA score but was associated with a varied range of gRNA scores (Supplementary Fig. 5b). This difference is because the *H. sapiens* genome is large and has more multi-targetable regions compared to the *C. elegans* genome. In both cases, a substantial number of gRNAs of varied standard deviation and with no off-targets could be selected.

### Scanning for multi-targeting gRNAs and multiple targetable regions

Using PyRanges, we created intervals of user-defined size that are overlapping with gRNA candidates containing the Cas9 PAM pattern (3′-NGG-5′). Since this step is computationally intensive, we have implemented options to narrow down the search with minimum and maximum number of hits for a target window.

Analysis of the annotation of regions of the *C. elegans* and *H. sapiens* genomes that can be targeted by multi-targeting gRNAs indicated that a broad range of features including genes and gene regulatory elements are available for selection. The range of annotated, targetable regions for each genome could be further significantly increased when combining gRNA searches for different genome-targeting systems that use different PAM sequences (Supplementary Fig. 6).

### Targeting of a broad genome region using CRISPR-broad

**Figure 7:**
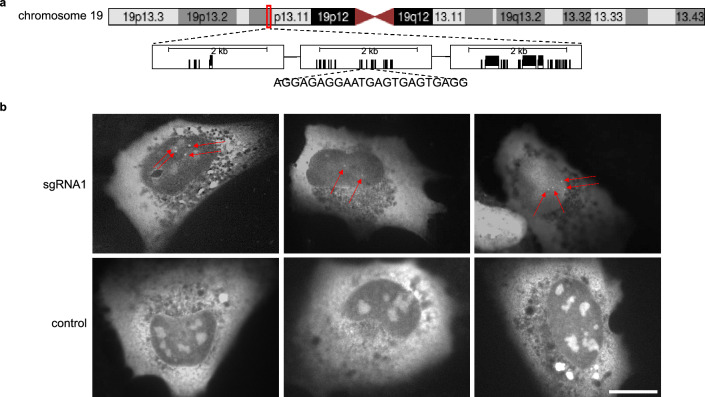
Targeting of a broad region of the genome using a singular gRNA designed by CRIPSR-broad. (**a**) Scheme depicting a 317 kb region on human chromosome 19 that can be targeted by a sgRNA at 86 locations. (**b**) Fluorescence imaging of U2OS cells transfected with a plasmid expressing dCas9-3XGFP together with a plasmid expressing the sgRNA targeting the region depicted in (A) (*top*) or the corresponding empty vector (*bottom*). Focal enrichment of GFP inside the nucleus is marked by arrows. Note that due to the different cell cycle stages two (2n chromosome content, G1- , S-phases) or four (4n chromosome content, G2-phase) labeled spots are expected. Scale bar represents 20 μm. Details on the selection of the presented cells and images can be found in Supplementary Fig. 8.

To test CRISPR-broad we resorted to a previously described method of “painting” genome regions by targeting dCas9 fused to green fluorescent protein (GFP). Singular gRNAs targeting more than 100 directly repeated sequences within telomeres or pericentromeres identified by classical gRNA design tools has enabled mapping of these functional chromosome elements in cellular context^4–6^. Using CRISPR-broad we identified a singular gRNA targeting a 317 kb region on human chromosome 19 at 19p13.2 with 86 hits (Fig. 7a). Human U2OS transfected with a plasmid expressing dCas9-3XGFP together with a plasmid expressing the identified sgRNA showed two or 4 dots of accumulated green fluorescence in the nucleus in agreement with a 2n (G1- and S-phase) or 4n (G2-phase) chromosome content. In contrast and as described before^4–6^, dCas9-3XGFP in the absence of specific gRNA-mediated targeting displayed nucleolar background staining in the cell nucleus (Fig. 7b). The results indicated that CRISPR-broad can identify large genomic regions for efficient targeting of dCas9 apart from simple and obvious repetitive elements of the genome.

### Overlap of target regions with epigenetic features

To assess the wider application and potential of CRISPR-broad, we compared the results of the test runs on the *C. elegans* and *H. sapiens* genomes using the single Cas9 PAM with annotated (epi-)genetic features using the ENCODE and modENCODE datasets. We found the multi-targetable windows defined by CRISPR-broad overlapping with the features transcription factor binding sites (ChIP-seq peak regions), histone modification region (ChIP-seq peaks), annotated transposable elements in the genome and sites of DNA methylation (WGBS: methylated CpG sites). The fact that the fraction of each of these sites that could be targeted by multi-targeting gRNAs (number of features overlapped to a gRNA window of 5 kb/total number of features) is substantial (Supplementary Fig. 7) indicated that CRISPR-broad could be useful in various strategies of epigenome editing.

### Performance

CRISPR-broad was developed in Python and the source code is available at https://github.com/AlagurajVeluchamy/CRISPR-broad. CRISPR-broad runs in seven independent modules with multiple options for user input (Fig. 1b). The limiting steps are mapping the gRNAs to the genome and obtaining all hits. We tested the performance of the tool on a Linux workstation with 30–40 threads computed for genome sizes of 103 Mb (*C. elegans*) and 3.2 Gb (*H. sapiens*) (Table 1). With an increase in genome size and in the allowed number of mismatches, the run time increased. The gRNA sequences, aggregate gRNA scores, GC content, number of on- and off-target hits, optimal on-target window of pre- selected size, and co-ordinates of each hit are compiled and exported in a tab-delimited text (Supplementary Table 2).

**Table 1:** Benchmark results of CRISPR-broad performance with multithreading in two datasets.

Species	Genome size	Pre-filtered gRNAs with PAM (NGG)	Candidate gRNAs (with at least 5 hits in a window)	Run time (min)
*C. elegans*	103 Mb	5,734,064	27,878	100
*H. sapiens*	3.2 Gb	242,662,048	2,413,602	360

Run time was deduced from multimapping of 10,000 genome-wide locations per gRNA for *H. sapiens* and without restriction for *C. elegans*. The number of gRNA targeting windows was calculated with three mismatches. The run time for the *C. elegans* genome corresponds to 30 threads with 50 kb windows, the run time for the human genome corresponds to 40 threads with 500 kb windows.

## Discussion

We introduce a gRNA design tool that is distinct from the “one-gRNA-one target” approach. Two model genomes, C*. elegans* and *H. sapiens* were scanned for multi-targeting gRNAs*.* We attribute the fast execution of the tool to the parallelizing strategy using a multiprocessing/multithreading approach and the application of Pandas-based big data analytics modules. GC content-based gRNA filtering further speeds up the gRNA design process. Given that CRISPR-broad identified regions in the model genomes that can be targeted by multi-targeting gRNAs of various annotation and (epi-)genetic features (Supplementary Figs. 6 and 7), we are confident the new tool could be useful in various experimental schemes:

### Fluorescence mapping of genome regions and their imaging in live cells

Our results show that CRISPR-broad can identify broad genome regions for mapping via dCas9 fused to fluorescent proteins. Combination of CRISPR-broad with more sensitive visualization methods that allow signal amplification such as the Sun-Tag system should enable cellular mapping of many and wide regions of selected genomes^30^. Indeed, it has been demonstrated that 24 copies of GFP recruited via the SunTag enables long term chromosome imaging in living cells^30,31^. We envision that the combination of multi-targeting gRNAs, multiplexed target regions, signal amplifying detection systems and deactivated nuclease systems relying on different PAM sequences could enable “live chromosome painting”, i.e. the simultaneous real time visualization of multiple genome elements during various cellular processes such as for example the cell cycle.

### Level of activation of transcription

Cellular activation of genes can be achieved by dCas9 fused to transcriptional activators such as p65-HSF1. The level of activation of target genes is directly correlated to the number of activation domains recruited to the promoter region. For example, to induce neuronal conversion of astrocytes in vivo efficiently, each neuronal factor gene (ANN: *Ascl1*, *Neurog2* and *Neurod1*) needed to be targeted by multiple sgRNA^32^.

### Targeting transposable elements or repeat regions

Transposon mutagenesis allows establishing gain of function or loss of function in cancer patients^33^. This is enabled by CRISPR-Cas induced genetic and epigenetic alterations. A CRISPR screen on transposons such as *Sleeping beauty* (SB) and *piggyBac* (PB) has been shown to provide insights into cancer initiation, progression, metastasis, and resistance to treatment^34^. Such mutagenesis strategy could be extremely simplified by multi-targeting gRNAs.

### Genome compartmentalization and organization studies

The recently developed CRISPR-GO system can efficiently control the spatial positioning of large genomic regions relative to specific nuclear compartments, TADs, and including the nuclear periphery^35^. The approach offers a programmable platform to investigate large-scale spatial genome organization when using multi-targeting gRNAs. Any large region in a genome could be segregated or positioned to study the role of genome compartmentalization and potentially nuclear bodies (e.g. Cajal bodies) using multi-targeting gRNAs.

### Large-scale induction of DNA methylation

Engineered DNA methyltransferases such as dCas9-MQ1 and dCas9-DNMT3a were shown to methylate CpG at sgRNA directed target sites^36,37^. Methylation induced by a pair of sgRNA decreases with increasing intervening distance between two CpG sites^13^. The number of CpG sites in genome regulatory regions such as CpG islands is large, and it is impossible to target these by singular gRNAs. Instead, a pool of sgRNAs is required^37^. This increases the chance of off-target effects and makes the experimental strategy more expensive and technically challenging. Multi-targeting gRNAs could resolve these problems.

### Investigating the role of histone modifications

Manipulating histone modifications in cellular context and in large genomic regions will help understanding their functional implications. For example, inducing the repressive modification H3K27me3 in *Oryzias latipes* by using the Ezh2 methyltransferase (olEzh2) fused to dCas9 was accomplished with multiple sgRNA^38^. Using multi-targeting gRNAs could simplify such approach and expand its scope.

We envision that CRISPR-broad will enable targeting the genome and epigenome at different levels of functionally and/or structurally annotated regions such as promoters, enhancers, CpG islands, multi-genes requiring longer coverage, topologically associating domains (TADs, 100 kb–1 Mb) and putatively compartments (several Mb). There are likely more applications for CRISPR-broad than the ones we outlined here, and we are looking forward to seeing how this tool will enable novel applications of the CRISPR-Cas and related RNA-targeted nuclease systems.

## Conclusions

In summary, we developed CRISPR-broad which enables users to define multi-targeting, unique gRNAs in conjunction with multiplex or expanded targeted genome regions. Our new strategy bypasses the need for identifying multiple individual sgRNAs or for relying on highly repetitive elements bound by singular gRNAs for targeting larger regions of a genome. Using CRISPR-broad, we demonstrated successful multi-targeting gRNA design for two model genomes, C*. elegans* and *H. sapiens*. Analyzing the two genomes with different window sizes, numbers of bins and for different genome-editing systems (i.e. using different PAM sequences), revealed that multiple potential target regions covered by multi-targeting sgRNAs are available for selection and irrespective of the repeat rich sequence content of each genome. Lastly, we verified the usability of CRISPR-broad by identifying a singular multi-targeting gRNA for visualization of a 317 kb region of the human genome using dCas9-GFP. We envision that CRISPR-broad could have wide applications in genome mapping and epigenome editing.

## Methods

### Step 1: Extraction of all candidate gRNA and all possible targets from a genome

The input genome FASTA is scanned for the user-defined parameters of specific PAM pattern and length of sgRNA. The PAM pattern should be at the 3’-end of candidate gRNA sequences of both forward and reverse DNA strands including overlapping sequences (e.g. N20-NGG for PAM NGG and 23 nt gRNA sequences). At this stage (Module 1), the user can also filter the gRNAs with specified GC percentages. Candidate gRNAs with the selected protospacer and PAM features are then mapped onto the genome using Burrows-Wheeler Aligner (BWA)^39^. In parallel, the genome is indexed, and candidate gRNAs are mapped with ‘end-to-end’ no seed string alignment applying a range of parameters including mismatches, insertion, and deletion.

### Step 2: Scanning multi-mapped gRNAs in sliding windows

Mapping candidate gRNA sequences is performed in a non-iterative search mode (− N) without seeding (− l). As short read mapping for off-targets is computationally expensive and time consuming, the module is separated to get mapped gRNAs from other tools. For user specified window sizes of k, CRISPR-broad filters singletons and creates a list of multi-mapped regions with information on the number of mismatches and gapped alignments. To detect genomic regions with most hits and of user defined size, a smaller sliding window based overlapping analysis is performed on each chromosome.

### Step 3: Check for PAM sequences in gRNA and computing scores for each window

User-defined PAM patterns are searched at the 3’-end of each of the target sequences and candidates with mismatches are removed. *W*_*i*_, a window score based on the matches and mismatches in a user-defined window size is calculated as follows:

$$W_{i} = \mathop \sum \limits_{i = 1}^{n} \left( {\frac{{m_{i} - n_{i} - g_{i} }}{L}} \right)$$

where $m_{i} , n_{i} , g_{i}$  and *L* correspond to the number of matches, the number of mismatches as well as the gap and length of a gRNA_i_. While the score is linearly proportional to the number of matches, mismatches and gaps in the alignments are penalized. We penalize mismatches of the nucleotides equally, irrespective of the position relative to the PAM sequence. The score *S*_*i*_ (aggregate gRNA score) for gRNA_*i*_ is derived as follows:

$$S_{i} = E + \frac{h}{t} \times \mathop \sum \limits_{i = 1}^{n} W {-}\frac{o}{t} \times \mathop \sum \limits_{j = 1}^{k} W^{\prime}$$

where *E* is the evenness/distribution of the multi-mapped guide RNAs in the target window, *h* is the number of hits in the same on-target window as the gRNA, *t* is the total number of hits, and *o* is the number of off-target hits (alignments that fall outside the user-specified or on-target window).

### Step 4: Scanning multi-targeting gRNAs and multiple targetable bins

For each candidate gRNA, genomic intervals of user-defined number and size of target bins are created using PyRanges based overlap analysis. The number of off-targets is derived from the table containing candidate gRNA with PAM patterns initially created in Step 1.

### Step 5: Pooling more than one gRNA in a target region

Multiple gRNAs can be pooled, and windows can be filtered that are targeted by specific and user-defined gRNAs. The output of Step 4 is aggregated and filtered. gRNAs are pooled according to Bedtools based overlapping analysis. Filtering of the gRNAs that fall within a window is performed using Python Pandas.

### Flexible input processing from user-defined regions

gRNAs can be searched in either singular, user-defined regions or within a set of coordinates. In this case, gRNAs are chosen from the user-defined region and off-targets are searched against the whole genome. Alternatively, de novo selection of gRNAs and ranking of all possible targetable regions can be done. In either case, entire genomes should be provided as input.

### Scanning for gRNAs on two different model genomes

Identification of potential gRNA candidates was performed on two different model systems, the 103 Mb genome of *Caenorhabditis elegans* (*C. elegans*) and the 3.2 Gb genome of *Homo sapiens* (*H. sapiens*). In doing so, different parameters of CRISPR-broad were tested and benchmarked. Annotation of the target regions of gRNAs were performed using annoPeak^40^. The spectrum of genome regions that can be accessed by multi-targeting was evaluated by selecting gRNAs with different PAM patterns. Besides the on-target hits, CRISPR-broad lists gRNA alignments that fall outside the user-specified window as off-targets. Off-targets were predicted for all gRNAs for both *H. sapiens* and *C. elegans* genomes with the PAM pattern NGG. To verify the CRISPR-broad scoring, we used an independent off-target tool, CasOffinder^19^.

### Genome mapping using multi-targeting gRNAs

Plasmid pHAGE-TO-dCas9-3XGFP expressing dCas9-3XGFP (#64107) and a sgRNA expression vector (#68463) were obtained from Addgene. Oligonucleotides corresponding to the identified multi-targeting gRNA (forward 5′-*GGAC*GAGGAGAGGAATGAGTGAGTG-3′, reverse 5′-*TTTC*CACTCACTCATTCCTCTCCTC-3′) were cloned into the BbsI sites of the sgRNA expression vector.

U2OS cells were cultured in DMEM (Life Technologies) supplemented with 10% (v/v) FBS and maintained at 37 °C. 300,000 cells were seeded on laminin coated microscopy cover slips in 6-well dishes and transfected with 150 ng of dCas9-3XGFP and 750 ng of sgRNA plasmid using Lipofectamine LTX (Life Technologies). After 24 h cells were washed in PBS and fixed with 2% formaldehyde in PBS.

Fluorescence microscopy imaging was done on a Leica Thunder system at 470/28 nm excitation and 512/23 nm emission wavelength. Imaging data were acquired with LAS X Software (Leica) and analyzed using Image J software.

### Supplementary Information

Supplementary Information.

## Data Availability

The source code of CRISPR-broad can be found at: https://github.com/AlagurajVeluchamy/CRISPR-broad. Documentation on installation and execution are available at this source. Operating system(s): Platform independent. Programming language: Python 3.X. Other requirements: bwa-0.7.11, Python 3.9.1, biopython 1.78, pandas 1.2.0 pyranges-0.0.95. License: GNU GPL 3.0. Any restrictions to use by non-academics: None.
