# Supplementary material for: CRISPR-broad: combined design of multi-targeting gRNAs and broad, multiplex target finding
Source: Sci Rep. 2023 Nov 12;13:19717. doi: 10.1038/s41598-023-46212-x (PMC10641073; doi:10.1038/s41598-023-46212-x)
Supplement: Supplementary file 1 — Supplementary Information. [file 41598_2023_46212_MOESM1_ESM.pdf]

Supplementary Material

CRISPR-broad: combined design of multitargeting gRNA and broad, multiplex target finding

Alaguraj Veluchamy[1,2], Kaian Teles[1] and Wolfgang Fischle[1]

[1] Bioscience Program, Division of Biological and Environmental Sciences and Engineering, King Abdullah University of Science and Technology (KAUST), Thuwal 23955-6900, Kingdom of Saudi Arabia.

[2] Department of Computational Biology, St. Jude Children's Research Hospital, Memphis, TN, USA.

Supplementary Figures

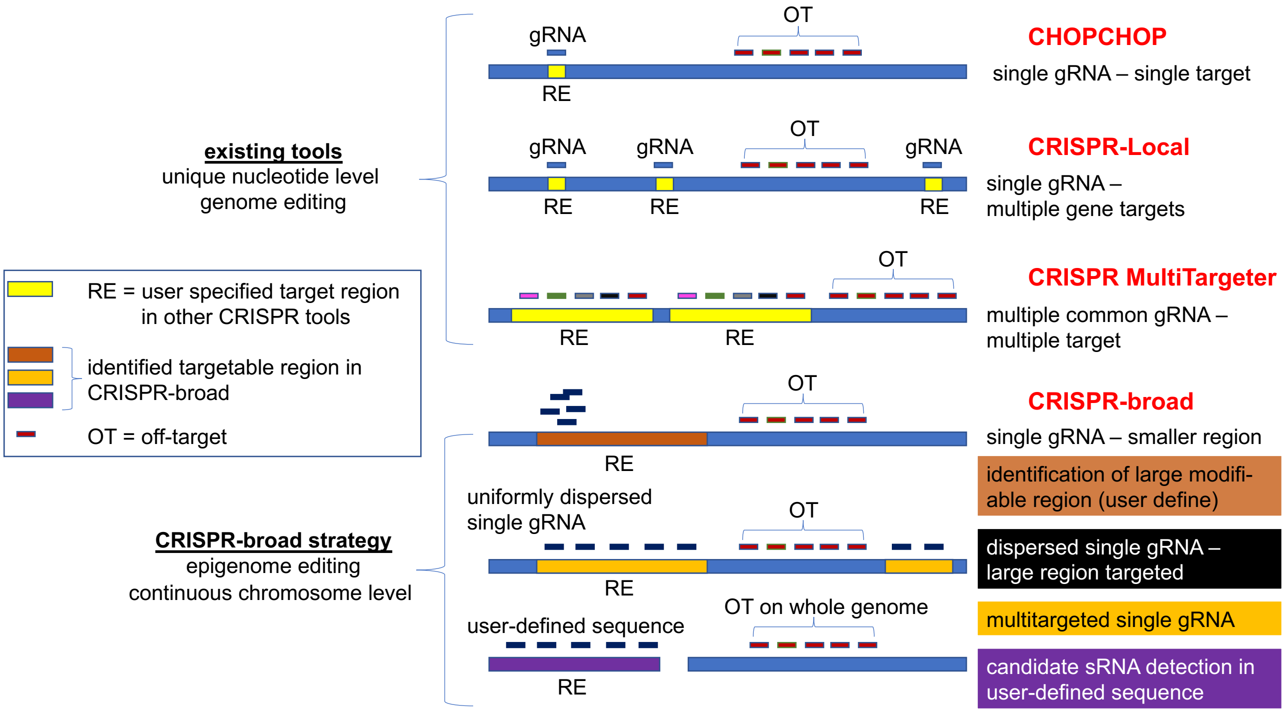

Supplementary Figure 1: ***Strategy of the CRISPR-broad tool compared to existing approaches for CRIPR-Cas gRNA design.*** Tools such as FlashFry, CRISPOR, CRISPR-DO, CasFinder, sgRNAScorer2, TUSCAN and CHOPCHOP apply a "one-gRNA to one-target" approach. While CRISPR-Local and CRISPR MultiTargeter can target several regions, these tools are not suitable for targeting specific broad regions of a genome. CRISPR-broad, in contrast, finds gRNAs that have multiple targets dispersed in a defined section (user-defined size) of a genome without any off-targets. At the same time, CRISPR-broad identifies broad targetable regions (several targets for a single gRNA) in a genome.

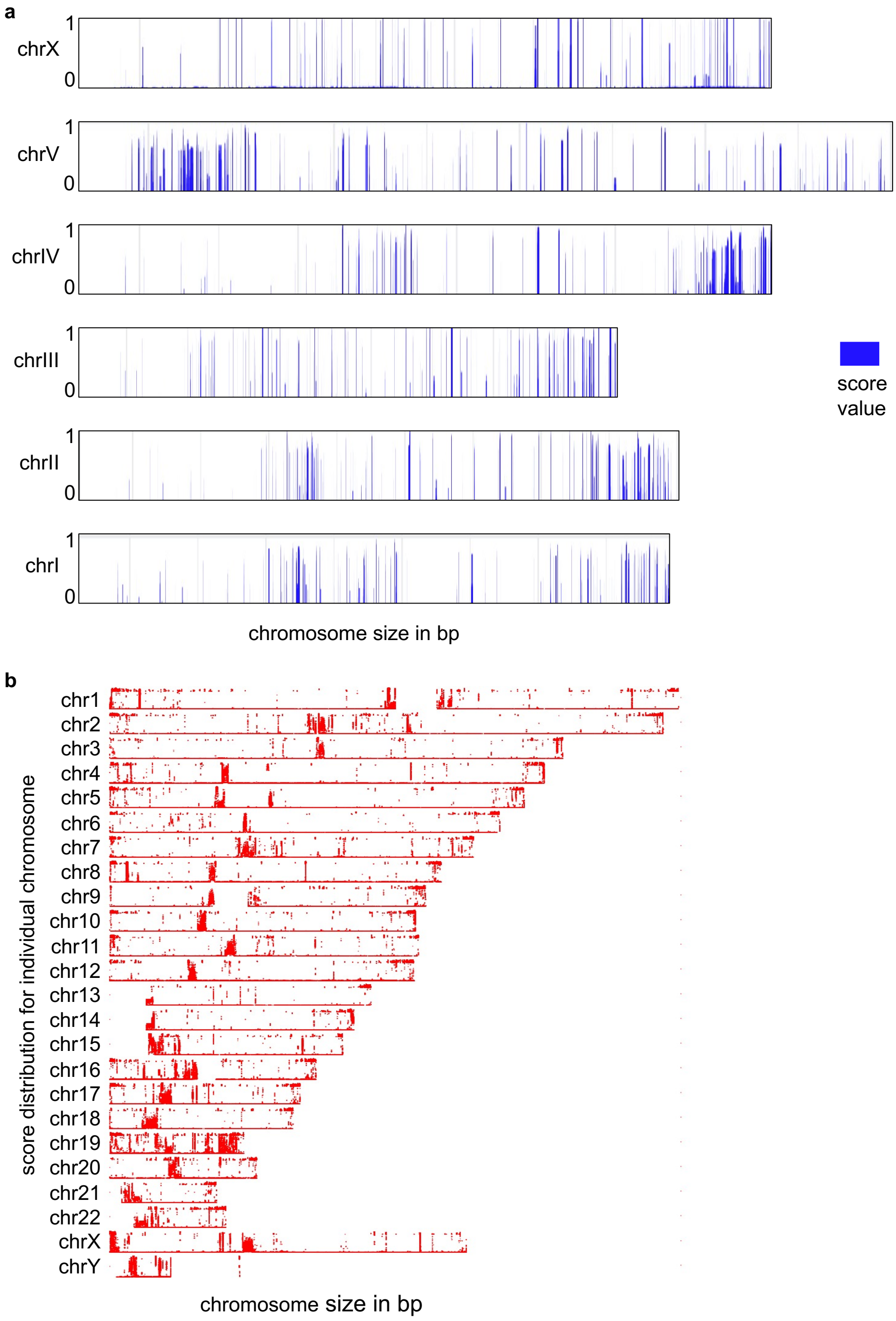

Supplementary Figure 2: *Distribution of gRNA sequences.* (**A**) *C. ele*gans chromosomes are plotted with data from a scan of gRNAs using a 50 kb window size. Blue lines indicate the gRNA hits. The aggegate gRNA score for each window is plotted. Only positive values are annotated, as these represent usable sgRNAs (**B**) *H. sapiens* chromosomes are plotted with data from a scan of gRNAs using a 500 kb window size. The GC content of gRNAs was set at 50%. GC based filtering of gRNA hits reduces the amount of data to be processed, since there are many repetitive, GC-rich sequences. Positive and higher aggregate gRNA scores correspond to regions which have fewer off-targets (y-axis reflects gRNA score ranging from 0 to 1 and X-axis represents length of chromosome in bp).

a

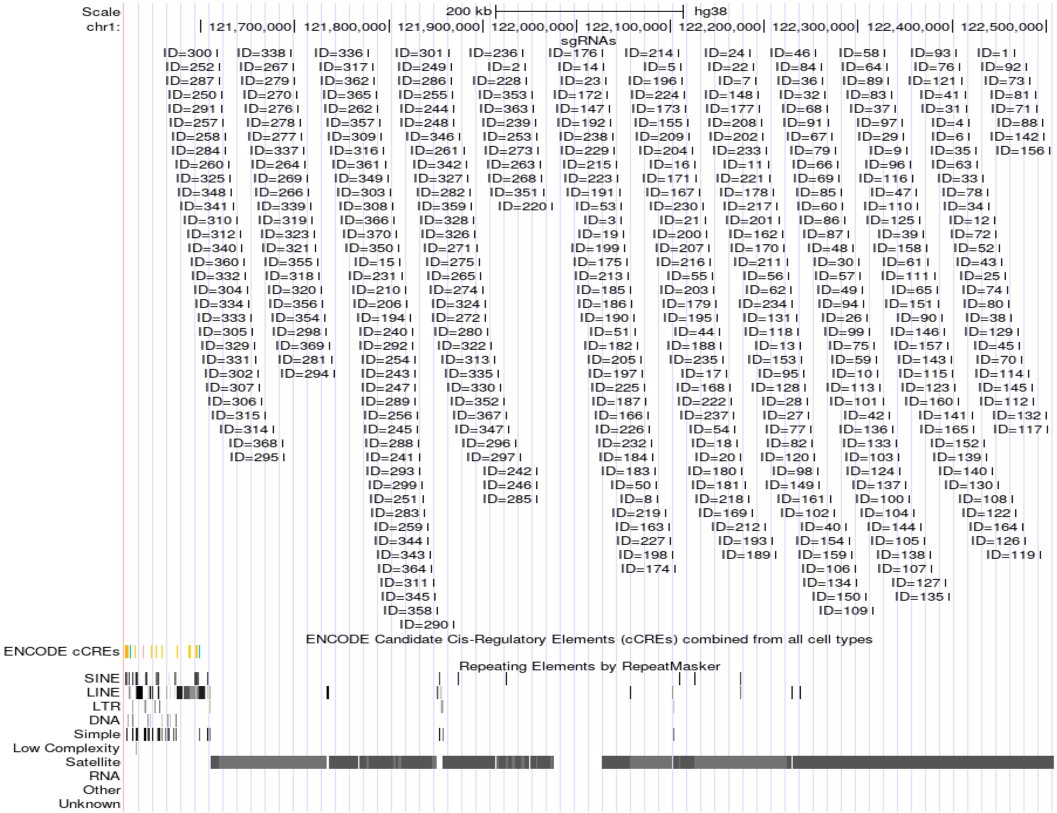
Scale
200 kb
hg38
chr1:
121,700,000
121,800,000
121,900,000
122,000,000
122,100,000
122,200,000
122,300,000
122,400,000
122,500,000
sgRNAs
ENCODE cCREs
ENCODE Candidate Cis-Regulatory Elements (cCREs) combined from all cell types
Repeating Elements by RepeatMasker
SINE
LINE
LTR
DNA
Simple
Low Complexity
Satellite
RNA
Other
Unknown

b

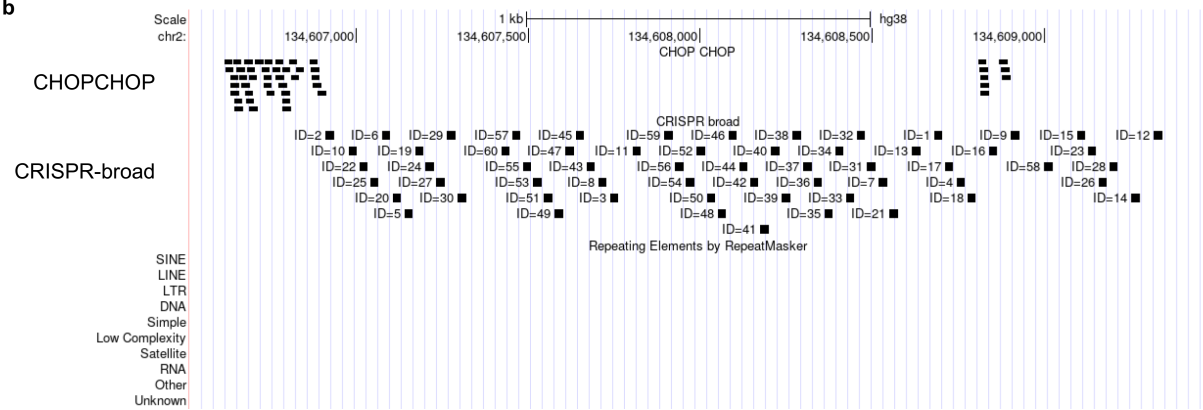
Scale
1 kb
hg38
chr2:
134,607,000
134,607,500
134,608,000
134,608,500
134,609,000
CHOP CHOP
CHOPCHOP
CRISPR broad
CRISPR-broad
Repeating Elements by RepeatMasker
SINE
LINE
LTR
DNA
Simple
Low Complexity
Satellite
RNA
Other
Unknown

c

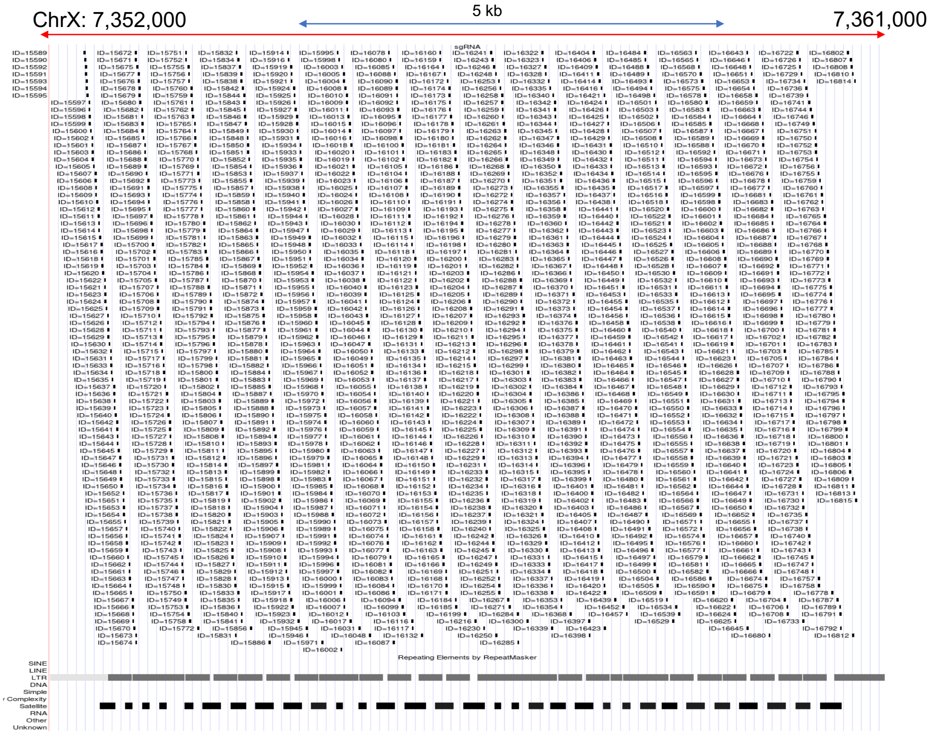

d

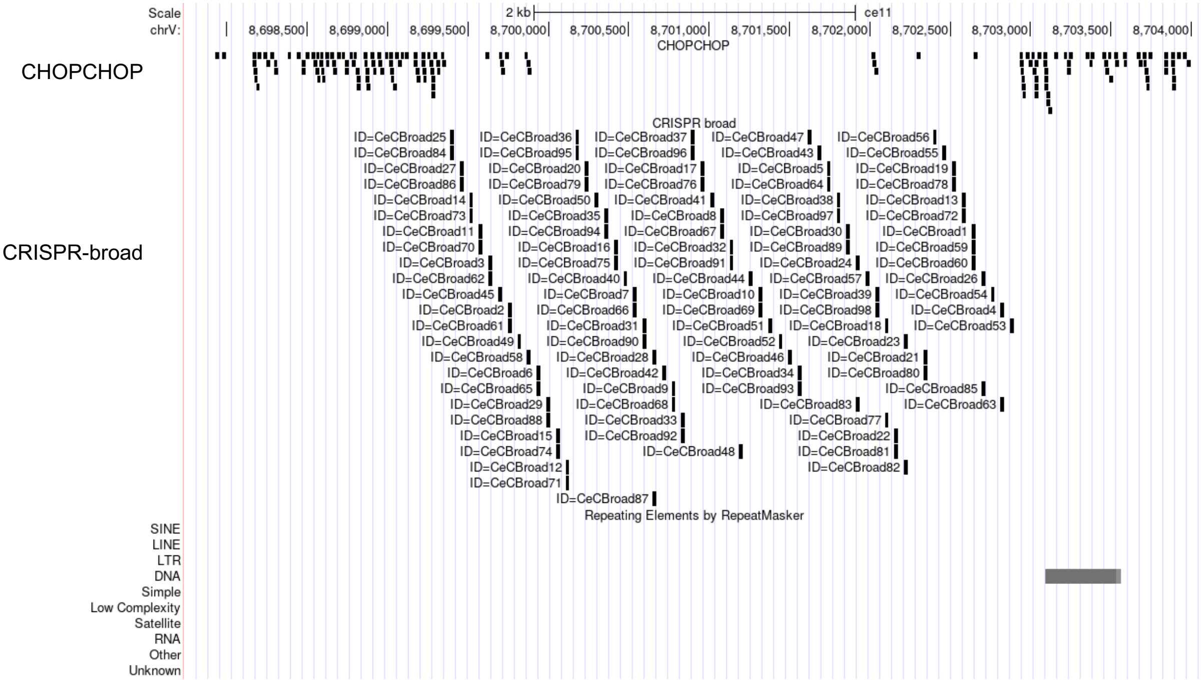

Supplementary Figure 3: ***Dispersion of gRNA hits.*** (**A** and **B**) Screenshot showing the dispersion of sgRNA hits identified with CRISPR-broad within a single bin in the pericentromeric region of chr1 (A) of chr2 (B) of *H. sapiens*. (**C** and **D**) Dispersion of sgRNA hits within a single bin in the pericentromeric region of chrX (C) and chrV (D) of *C. elegans*. gRNA labels indicate the number of hits in the alignment for a particular gRNA. High standard deviation from the middle of the selected bin (target window) shows even distribution of the candidate gRNA. For (B) and (C) targeting of the same regions as identified by CHOPCHOP is indicated and the scan of the region by RepeatMasker is given.

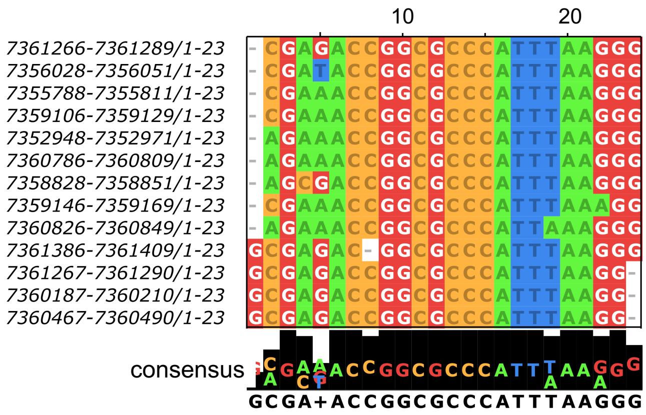

Supplementary Figure 4: ***Analysis of mismatches.*** Each gRNA from *C. elegans* was pairwise aligned to the target window in the genome assembly Ce235 with different levels of mismatches using short read aligner. Mismatches around the PAM sequences are anti-correlated to the specificity and effectiveness of a gRNA. Nucleotide mismatches are distributed throughout the alignment position from 5' to 3' of sequences. Each of the 23 nt nucleotide sequences show varied levels of mismatches. This particular sgRNA has a score value of 0.8.

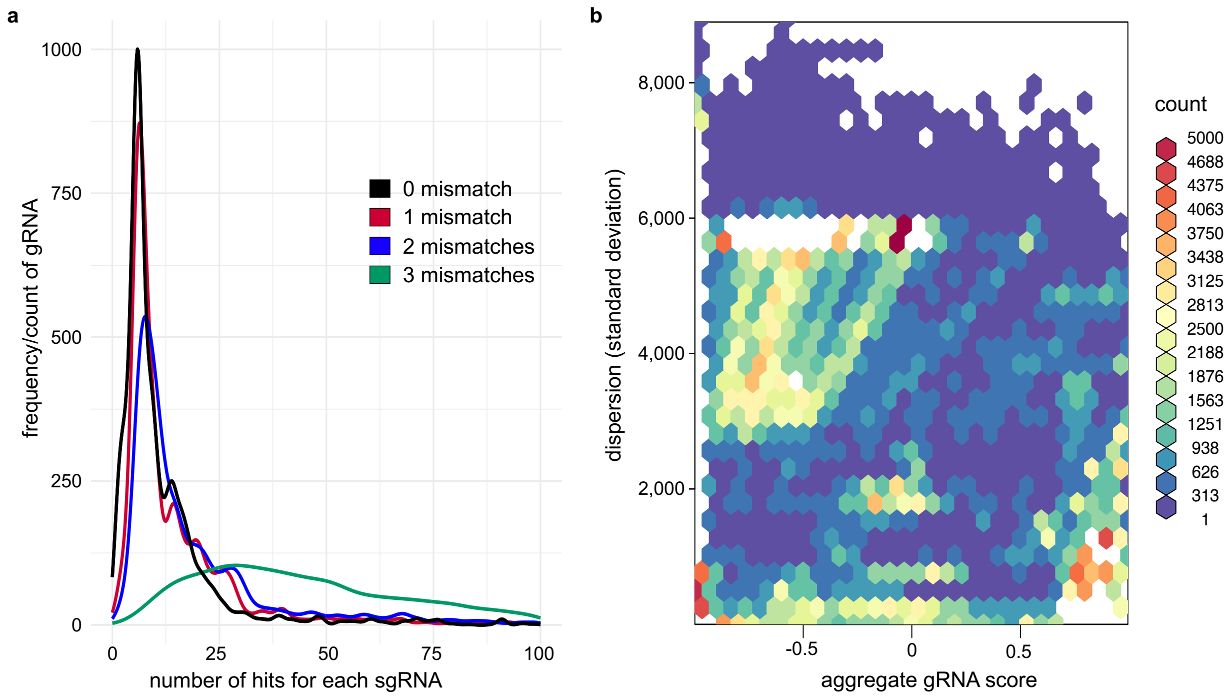

Supplementary Figure 5: ***Efficiency of CRISPR-broad.*** (**A**) CRISPR-broad was used to randomly scan 10,0000 potential gRNAs for different levels of mismatches in *H. sapiens*. Mismatch levels were set in the range from 0 to 3. Earlier reports have shown that gRNA efficiency is affected by the level of mismatches. The subsampling shows that a significant number of gRNAs with few mismatches are available for selection. (**B**) Hexbin plot showing the relationship between aggregate gRNA score and dispersion. Standard deviation (dispersion) was calculated from the position of the gRNA hits within a target window. The aggregate gRNA score ranges from negative to positive values. Higher values of standard deviation correspond to higher distribution of gRNA within a target window. Standard deviation and gRNA score were calculated using 500 kb windows in *H. sapiens.*

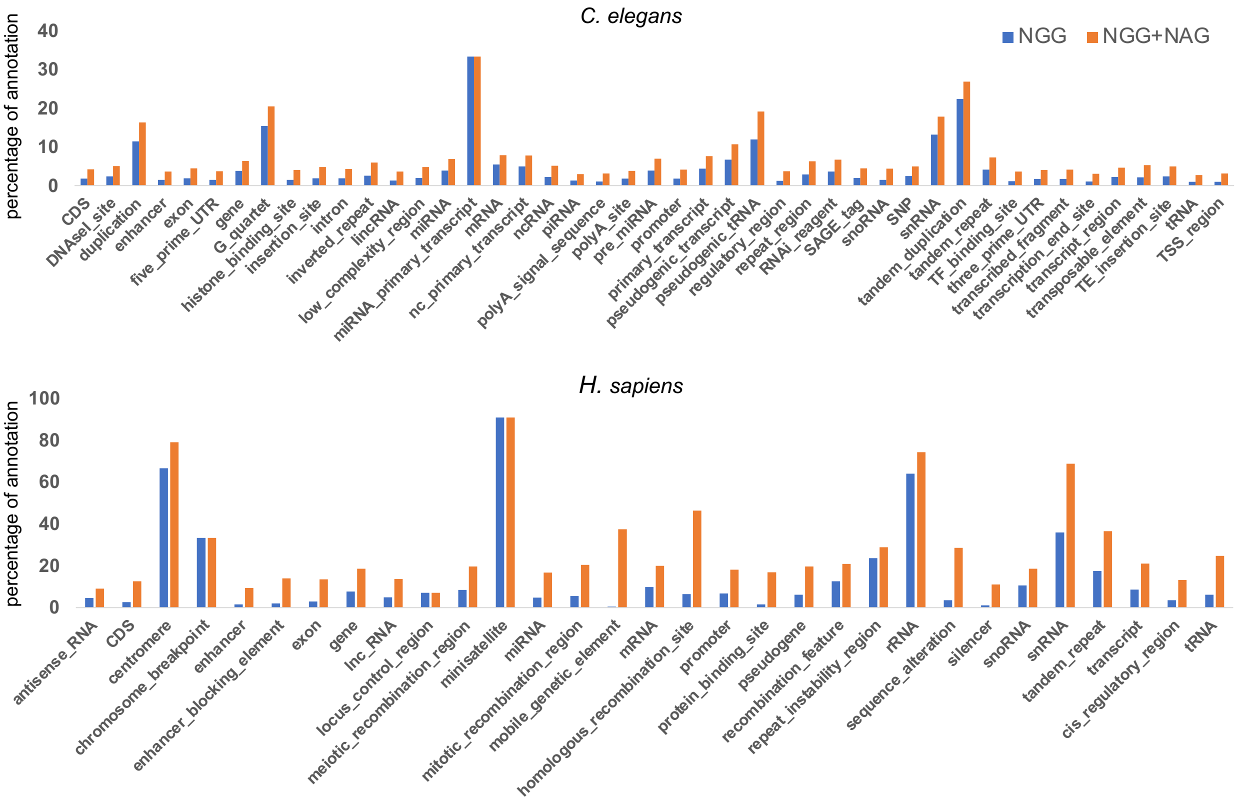

Supplementary Figure 6: ***Annotation of targetable regions that were selected by CRISPR-broad.*** Barplot showing the percentage of regions targetable with a unique multitargeting gRNA. The analysis was performed for the Cas9 editing system alone (PAM sequence NGG, *blue*) and a combination of the Cas9 and a non-canonical, less-preferred genome editing system (PAM sequences NGG and NAG).

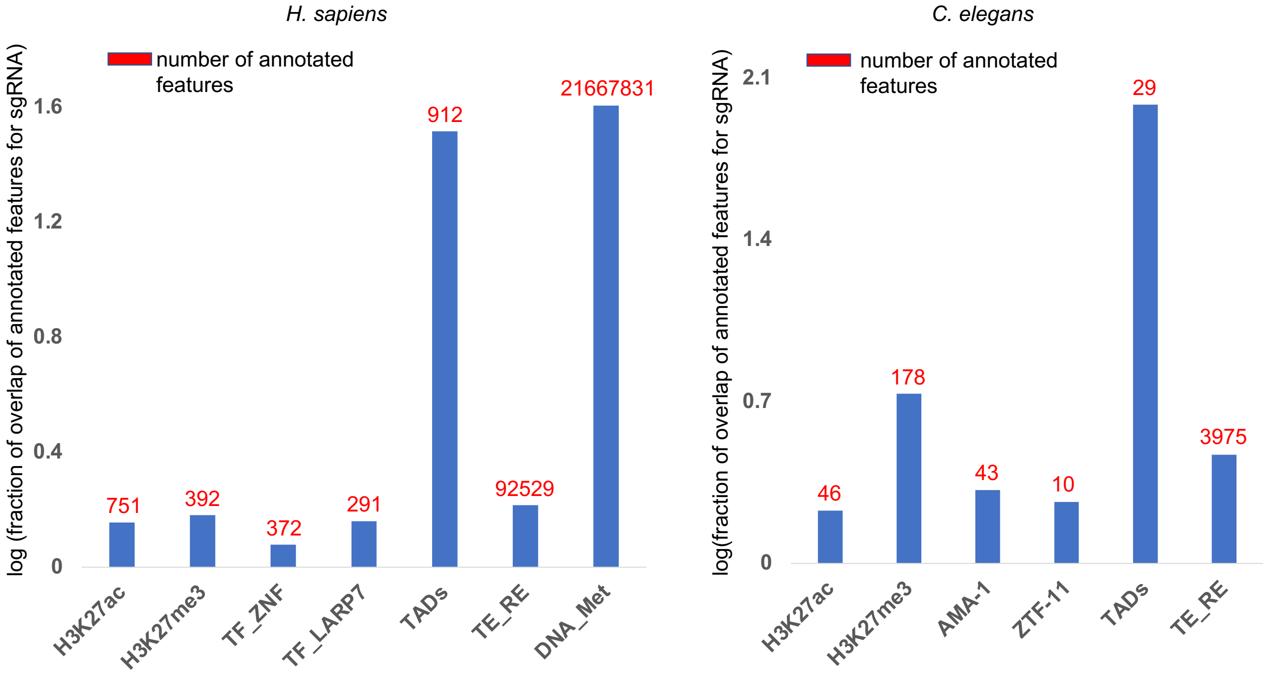

Supplementary Figure 7: ***Features (regions) targetable by CRISPR-broad.*** These are annotated by different experimental schemes: regions carrying histone modifications (ChIP-seq: H3K27ac, ChIP-seq: H3K27me3), DNA methylated regions (CpG methylation by WGBS: DNA_Met), transcription factor binding sites (selected examples: TF_ZNF, TF_LARP7, AMA-1, ZTF-11), topologically associating domains (TADs), transposable elements and repeat regions (TE_RE). Histone modifications and DNA methylation data are from ENCODE GM12878 for *H. sapiens* and modENCODE L3 stage for *C. elegans*. Barplot shows the overlap of each of these annotated regions (number and fraction of regions targetable with multitargeting gRNAs with a unique, single PAM). Numbers on top of the bars represent the total targetable regions; bars represents the fraction of annotatable regions. The analysis was performed for the Cas9 (PAM sequence NGG) alone with same window size, mismatches, GC percentage described in the methods section 2.1 and 2.3.

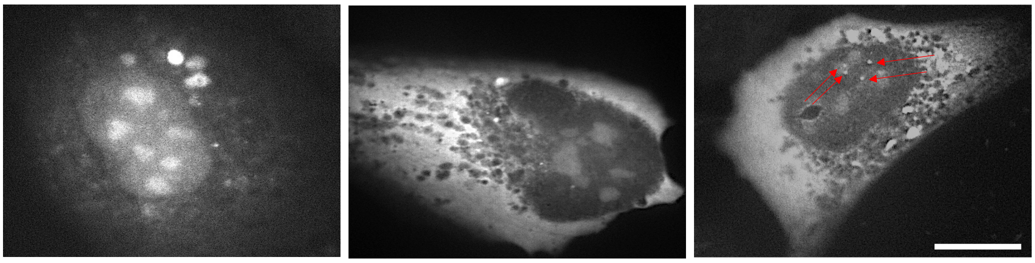

Supplementary Figure 8: ***Variation of fluorescent signal of cells transfected with gRNA for targeting a broad region of human chromosome 19 (related to Figure 7).*** U2OS cells representative of the different phenotypes and fluorescent signals observed after transfection with plasmids expressing dCas9-3XGFP and the sgRNA depicted in Fiugure 7 are shown. Many cells showed flourescent signal in the nucleolus (left) or the cytoplasm and nucleolus (middle). These might not have been transfected with the gRNA expressing vector. Nonethelss, a significant number of cells showed punctuated staining in the cell nucleus as indictaed by the arrows for the cell on the right and as predicted for targeting of dCas9-3XGFP to the singular broad region on chromsosome within 19p13.2. Scale bar represents 20 μm.

Supplementary Tables

Supplementary Table 1: ***Comparison of tools for CRISPR gRNA design. Both web-based and standalone tools are available.***

tool name	category	feature	choosing one/multiple best genomic window(s)	whole genome
CHOPCHOP	target	one gRNA – one target	no	40 kb max for web-version
CRISPRdirect	target	one gRNA – one target	no	10 kb for web-version
CRISPR-DO	target	one gRNA – one target; no off-target information; emphasis on specificity	no	for web-version
CRISPOR	off-target	one gRNA – one target	no	2 kb or pre-selected window
Cas-OFFinder	off-target	23nt gRNA needed to detect off-targets	no	23 nt gRNA needed
CRISPR MultiTargeter	target	one gRNA – two targets (finds common and unique gRNA)	no	50 kb
CRISPR-broad	target	one gRNA – one window	yes	yes

The tools differ in their function for scanning of single gRNAs that can target a particular region of a genome or for eliminating putative, multiple off-targets. All previously available tools are limited in their capability to scan large regions or whole genomes for gRNAs. Cas-Offinder and other tools require defined gRNAs as input to enumerate off-target hits.

Supplementary Table 2: ***Output of the CRISPR-broad tool.***

candidate gRNA	gRNA sequence	GC %	score	number of hits in best window	number of hits outside best window	best window
crnafs_X_i203952_7351479	AGCGATTCCTTACCCTTAAATGG	43.48	0.989	230	0	X:7351457-7361335
crnars_X_i682195_7351464	CCGGCGCCCATTTAAGGGTAAGG	60.87	0.985	247	0	X:7351464-7361342
crnars_II_i745926_9808150	ATGGAGTTTCAATTGTGCACTGG	43.48	0.975	291	1	V:9807972-9825305
crnafs_IV_i21067_688912	TGGACGATCAATTCCATCTTTGG	43.48	0.972	91	0	IV:688788-698168
crnafs_IV_i197230_6683650	TTCAAGTTTTTTGAAATAACTGG	26.09	0.968	263	0	IV:6668375-6731733
crnars_X_i682262_7352349	CGAAACCGGCGCCCATTTAAGGG	60.87	0.966	246	0	X:7351469-7361347
crnars_II_i606550_4641346	TCCATATTGTCCATAAATCTCGG	39.13	0.965	59	0	II:4641288-4644953
crnafs_IV_i197254_6685780	TAATGATATGTACAAACGCATGG	34.78	0.963	99	0	IV:6668679-6731783
crnars_IV_i498494_689344	TCGACATATCAAGGTGGTACTGG	47.83	0.962	51	0	IV:688834-698112
crnafs_IV_i197288_6688227	TGTGTATATGTACAGACGCATGG	43.48	0.959	159	0	IV:6668335-6731052

The *C. elegans* genome was scanned for gRNA candidates containing the 3’-NGG-5’ PAM pattern of Cas9 on a window size of 50 kb. The maximum allowed mismatches were kept at 3. The top scoring 50 kb windows with high aggregate gRNA score and minimum off-targets were ranked and tabulated. Candidate gRNAs were given a name with a naming convention fs = forward strand, rs = reverse strand, n = unique number for crna candidate and last number in the gRNA name corresponding to candidate chromosomal window. Number of hits in best window refers to multiple onN-target hits and number of hits outside best window refers to off-target hits.
